# Temperature affects organic acid, terpene and stilbene metabolisms in wine grapes during postharvest dehydration

**DOI:** 10.3389/fpls.2023.1107954

**Published:** 2023-01-30

**Authors:** Ron Shmuleviz, Alessandra Amato, Mauro Commisso, Erica D’Incà, Giovanni Luzzini, Maurizio Ugliano, Marianna Fasoli, Sara Zenoni, Giovanni Battista Tornielli

**Affiliations:** Department of Biotechnology, University of Verona, Verona, Italy

**Keywords:** postharvest dehydration, vitis vinifera, temperature, stilbene metabolism, terpene metabolism, grape

## Abstract

The partial dehydration of grapes after harvest is a traditional practice in several winegrowing regions that leads to the production of high quality wines. Postharvest dehydration (also known as withering) has a significant impact on the overall metabolism and physiology of the berry, yielding a final product that is richer in sugars, solutes, and aroma compounds. These changes are, at least in part, the result of a stress response, which is controlled at transcriptional level, and are highly dependent on the grape water loss kinetics and the environmental parameters of the facility where grapes are stored to wither. However, it is difficult to separate the effects driven by each single environmental factor from those of the dehydration rate, especially discerning the effect of temperature that greatly affects the water loss kinetics. To define the temperature influence on grape physiology and composition during postharvest dehydration, the withering of the red-skin grape cultivar Corvina (*Vitis vinifera*) was studied in two conditioned rooms set at distinct temperatures and at varying relative humidity to maintain an equal grape water loss rate. The effect of temperature was also studied by withering the grapes in two unconditioned facilities located in geographic areas with divergent climates. Technological, LC-MS and GC-MS analyses revealed higher levels of organic acids, flavonols, terpenes and cis- and trans-resveratrol in the grapes withered at lower temperature conditions, whereas higher concentrations of oligomeric stilbenes were found in the grapes stored at higher temperatures. Lower expression of the malate dehydrogenase and laccase, while higher expression of the phenylalanine ammonia-lyase, stilbene synthase and terpene synthase genes were detected in the grapes withered at lower temperatures. Our findings provide insights into the importance of the temperature in postharvest withering and its effect on the metabolism of the grapes and on the quality of the derived wines.

## 1 Introduction

Grapevine is one of the most important plant species especially due to the economic impact of its processing that leads to a wide range of wines largely traded and consumed worldwide. To meet certain wine styles, grape clusters can be left on the plant beyond ripening, or can be harvested and stored in dedicated dehydrating rooms prior to winemaking. In some wine regions such as the Verona province (Northeastern Italy), the latter process is followed to obtain Amarone, a premium wine largely commercialized all over the word. Grape postharvest dehydration (also known as withering) is a dynamic process that can last up to 90-100 days and features water loss and concentration of sugars and other metabolites, impacting both alcohol content and sensorially and technologically relevant wine constituents such as phenolics, aroma compounds and aroma precursors. Besides solutes concentration, metabolic processes already in play or specifically activated during withering may affect berry composition at the end of the process (Costantini et al., 2006; Mencarelli et al., 2010). The several physiological and biochemical changes described in postharvest berries were shown to be affected by factors like the environment and the genotype (Zenoni et al., 2016; Zenoni et al., 2020). In addition, structural modifications in the cell wall, likely influencing the extractability of solutes in the must during winemaking, were reported (Zoccatelli et al., 2013; Fasoli et al., 2019; Medina-Plaza et al., 2022). The transcriptional and metabolic reprogramming occurring during the process was also largely investigated at transcriptomic level in several works. These studies revealed that a massive gene modulation occurs in post-ripening berries and, at least in part, is responsible of the improved quality traits of withered grapes (Zamboni et al., 2008; Rizzini et al., 2009; Fasoli et al., 2012; Zenoni et al., 2016). For example, Zenoni et al. (2016) showed that an increase in sesquiterpenes and balsamic monoterpenes, contributing to the final aroma of Corvina wine (Slaghenaufi and Ugliano, 2018), occurs during postharvest dehydration of cv. Corvina grapes, in parallel to the upregulation of several members of the terpene synthase gene family. Similarly, it was observed that the accumulation of stilbene compounds followed the remarkable upregulation of genes encoding phenylalanine ammonia-lyases, stilbene synthases and laccases, in addition to cell wall remodeling expounded by the induction of pectin methylesterase encoding genes (Zoccatelli et al., 2013; Zenoni et al., 2016).

It is also known that the above-described changes largely depend on the environmental parameters (e.g., temperature, relative humidity, air flow and light) at the dehydration facilities that can have a profound impact on some grape characteristics such as skin force, polyphenolic composition, volatile organic compounds profile and on the final wine quality (Bellincontro et al., 2004; Rolle et al., 2013; Torchio et al., 2016; Tomasi et al., 2021). Grapes dehydrated under different environmental conditions generally differ in their dehydration kinetics and undergo increased water loss rate at higher temperatures and lower relative humidity (RH) (Barbanti et al., 2008). Noteworthy, Zenoni et al. (2020) recently investigated the specific effect of increasing dehydration rate with no temperature manipulation in grapes that exhibited – although mitigated – the typical postharvest transcriptomic program, indicating that the dehydration rate itself can promote compositional changes in the berries and then contribute to the final wine characteristics. Thus, when grapes are dehydrated at different temperatures it is excessively complex to define the extent of the specific contribution of either the temperature regime or the dehydration rate or their interaction to the observed effects on the grapes.

Our experimental plan aimed at dissecting the effect of temperature from other variables during the postharvest dehydration of grapes. We compared the composition of berries and wines from cv. Corvina grapes dehydrated at two temperatures, while maintaining the same water loss kinetics by adjusting the relative humidity conditions of the dehydration chambers. The temperature effect was also evaluated by monitoring grape withering in two unconditioned dehydration rooms featuring the natural environmental conditions of two different geographical locations in the Valpolicella growing area (Verona, Italy). The metabolomic analysis of volatile and non-volatile compounds, together with the expression analysis of selected genes revealed that the temperature has a direct impact on organic acids, phenolics and volatile compounds metabolisms, affecting important wine quality traits.

## 2 Materials and Methods

### 2.1 Experimental setup

Grape bunches of cv. Corvina (*Vitis vinifera* L.) were manually harvested at commercial ripening in season 2018 from vines grown in the property of the Allegrini Estate, located in the Valpolicella wine district (45°53’N; 10°83’E, 305 m a.s.l., Verona, northeast Italy). One layer of intact clusters (about 5-6 kg) was laid on perforated three plastic trays (40 x 60 x 15 cm each). The trays were placed for dehydration in two thermo-hygrometrically controlled rooms (16 m^3^). One room was set to higher temperature and relative humidity (Controlled High Temperature room, C-HT; 12 ± 0.5°C and 70 ± 3%) while the other room to lower temperature and relative humidity (Controlled Low Temperature room; C-LT; 8 ± 0.5 °C and 60 ± 2%). In both dehydration rooms the relative humidity was adjusted daily to maintain the same grape water loss (WL) rate and infer exclusively the temperature effect on the dehydration process. The rooms were kept under constant ventilation (0.2 m/s).

In the same season, grape bunches of cv. Corvina grown in the area of Gazzo di Marano di Valpolicella (45°55’N; 10°90’E, 361 m a.s.l.) were harvested in trays as describe above and split between one withering facility located in a plain area and one rather hilly to evaluate the temperature effect on dehydration process under natural conditions. The hilly withering facility located in Gazzo di Marano di Valpolicella (45°55’N;10°90’E, 382 m a.s.l.) registered the higher temperatures (Natural High Temperature room, N-HT), while the plain withering facility located in Valgatara (45°54’N; 10°91’E, 191 m a.s.l.) registered the lower temperatures (Natural Low Temperature room, N-LT). Temperature and relative humidity were monitored every two hours employing data loggers (HOBO UX100-023, Onset^®^, USA) during the withering process at all conditions (C-HT, C-LT, N-HT and N-LT).

### 2.2 Grape sampling and berry quality assessment

During the withering period, weight loss was determined by periodically weighting three trays placed in each condition to account for the biological replicates until the end of the dehydration process that was stopped at ∼25% and ∼30% berry weight loss (WL) for withering under controlled and natural conditions, respectively. Berries were collected from bunches at three time points corresponding to harvest time (T0), to ∼15% weight loss (T1) and at the end of withering (T2) (**Table S1**). For each sample, three replicates of 100 berries each were randomly collected. Only healthy undamaged bunches were considered for the analysis. The collected biological material was used for quality assessment of total soluble solids content (TSS; °Brix), titratable acidity (TA; g/L tartaric ac.), pH and molecular analysis (metabolomics and transcriptional analysis). The TSS, TA and pH were determined as previously described by Zenoni et al. (2016).

### 2.3 Metabolite extraction

Grape berry samples were differently managed depending on the dehydration stage as previously described (Brillante et al., 2018). For T0 samples, 300 mg of fresh frozen powders were weighed and extracted with 3 volumes (w/v) of methanol acidified with 1% (v/v) HCl. For T1 and T2 samples, 255 and 210 mg were respectively weighed and a volume of water equal to the measured mean weight loss occurred during withering was added. The extraction protocol was then applied like described for T0. The extraction was performed as previously described (Negri et al., 2021). Briefly, the extracts were vortexed for 30 s, sonicated at 40 kHz in an ultrasonic bath (SOLTEC, Milano, Italy) for 15 min in ice and centrifuged at 14,000× g at 4°C for 10 min. The supernatants were collected and eventually stored at -20°C. Prior the analysis, two quality controls were created by joining equal volumes from each sample belonging to a specific harvest year. Samples were diluted 1:10 with water LC-MS grade (Honeywell) and naringenin was used as internal standard at a final concentration of 100 pg/µL. The aqueous extracts were filtered through Minisart RC4 filters with 0.2 µm pores (Sartorius, Göttingen, Germany) and 2 µl were submitted to LC-MS analysis.

### 2.4 Untargeted metabolomics analysis

The instrument consisted of an ACQUITY UPLC I CLASS system (Waters, Milford, MA, USA) equipped with a reverse phase HSS T3 C18 column (2.1 mm × 100 mm, 1.8 µm), kept at 30°C. Water acidified with 0.1% (v/v) formic acid and pure acetonitrile were used as solvent A and B, respectively. The elution gradient started with 0% B; kept an isocratic condition at 0% B for 1 min; increased to 40% B at 10 min; reached to 70% B at 13.5 min; rose to 99% B at 14 min; kept an isocratic condition at 99% B for 2 min; decreased to 0% B at 16.10 min; kept an isocratic condition at 0% B (re-equilibrium to restore the initial condition) for 3.9 min. The method lasted 20 min. The flow rate was set at 0.350 mL/min. The instrument presented an autosampler Acquity FTN kept at 8°C, and an Acquity eLambda PDA detector (Waters). The mass spectrometer was a Xevo G2-XS qTOF mass spectrometer (Waters) with an electrospray ionization (ESI) as ion source set up with parameters described in a previous report (Commisso et al., 2019). Metabolites were ionized in either positive or negative ionization and MS data was acquired in continuum and sensitivity modes. The scan range was set to 50–2000 m/z, with a scan time of 0.3 s. CID fragmentation was performed by using Argon with a fixed collision energy set at 35 V. Few samples were analyzed twice by using a higher (45V) and lower (20V) fragmentation energy in order to facilitate the identification process. Metabolites identification was performed as previously described (Negri et al., 2021). The accuracy of the mass spectrometer was monitored by infusing a solution of 100 pg/µl leucine-enkephalin (flow rate of 10 µl/min) and generating a signal of 556.2771 in positive mode and 554.2615 in negative mode. Masslynx v4.1 (Waters) was used to control the UPLC-MS functions and to manually check the peak area of specific metabolite of interests. The MS data files were processed by using Progenesis QI (Waters), using default parameters, to get a data matrix including m/z features and the relative metabolite abundances.

### 2.5 Grape maceration and micro-vinification

At the end of withering, berries from three trays that were withered under the same conditions were pooled together and manually destemmed to obtain two batches of 12.5 kg each, respectively for the C-HT and C-LT rooms. To analyze the berry volatile compounds profile 500 g of berries were taken from each batch, in three replicates. The berries were then hand crushed with 50 mg of MBK and placed for maceration into 1- glass vessel where 100 ml of 15% (w/w) ethanol and 60 mg of dimethyl dicarbonate were added. The vessels were hand stirred daily for 8 days then pressed, clarified by centrifugation at 4500 rpm for 15 minutes at 5° C (Avanti J-25, Beckman Coulter, California, USA) and bottled in 330 ml glass bottles with crown caps.

As for micro-vinification, 3.5 kg berries were taken from each batch in three replicates. The grapes were then hand crushed with 100 mg/kg of potassium metabisulphite (MBK) and put into 5 L glass vessel. Glucose + fructose, total acidity and tartaric acid were analyzed using a Biosystems Y15 multiparametric analyzer (SinaTech Srl, Fermo, Italy). For must inoculation, 100 g of *Saccharomyces cerevisiae* Zinfandel commercial starter (Vason, Verona, Italy) were rehydrated in 1-l water at 37° C for 15 min and7.5 ml of the rehydrated culture was used to inoculate musts. Fermentations were carried out at 22 ± 1°C, with cap being broken twice a day by gently pressing it down skins with a steel plunger and density and temperature monitored daily. Upon completion of alcoholic fermentation (glucose + fructose < 2 g/l), wines were pressed, cold settled and then clarified by centrifugation at 4500 rpm for 15 minutes at 5° C (Avanti J-25, Beckman Coulter, California, USA). MBK was added up to a final free SO_2_ concentration of 25 mg/l, after which the wines were bottled in 330 ml glass bottles with crown caps.

### 2.6 Analysis of volatile compounds

For free and glycolyzed volatile compounds (VOCs) profile, solid-phase extraction (SPE) followed by GC-MS analysis was used, according to the procedure described by (Slaghenaufi et al., 2020). An amount of 100 µl of internal standard 2-octanol (4.2 mg/l in ethanol) was added to samples prepared with 50 ml of wine and diluted with 50 ml of deionized water. Samples were loaded onto a BOND ELUT-ENV, SPE cartridge (Agilent Technologies. Santa Clara, CA, USA) previously activated with 20 ml of dichloromethane, 20 ml of methanol and equilibrated with 20 ml of water. After sample loading, the cartridges were washed with 15 ml of water. Free VOCs were eluted with 10 ml of dichloromethane, and then concentrated under gentle nitrogen stream to 200 μl prior to GC injection. After SPE elution of free VOCs, glycosidic precursors were eluted with 20 ml of methanol and collected. Enzymatic hydrolysis was performed as described in Slaghenaufi et al. (2019). Glycosidic extracts were evaporated under vacuum thanks to Rotavapor (Buchi R-215 Rotavapor System), recovered with 5 ml of citrate buffer (pH 5), 100 mg of polyvinylpolypyrrolidone (PVPP) and 200 μl of enzyme solution AR2000 (70 mg/ml in citrate buffer) were added. Samples were stored at 37°C overnight. Aglycones were extracted as free volatile compounds with the SPE protocol as described above.

GC-MS analysis, was carried out on an HP 7890A (Agilent Technologies) gas chromatograph coupled to a 5977B quadrupole mass spectrometer, equipped with a Gerstel MPS3 auto sampler (Müllheim/Ruhr, Germany). Separation was performed using a DB-WAX UI capillary column (30 m × 0.25, 0.25 μm film thickness, Agilent Technologies) and helium (6.0 grade) as carrier gas at 1.2 ml/min of constant flow rate. GC oven was programmed as follows: started at 40°C for 3 min, raised to 230°C at 4°C/min and maintained for 20 min. Mass spectrometer was operated in electron ionization (EI) at 70 eV with ion source temperature at 250°C and quadrupole temperature at 150°C. Mass spectra were acquired in synchronous Scan (m/z 40–200) and SIM mode. Samples were analysed in random order. The quantification was done by calibration curves as described in (Luzzini et al., 2021). Calibration curve was prepared for each analyte using seven concentration points and three replicate solutions per point in model wine (12% v/v ethanol, 3.5 g/l tartaric acid, pH 3.5) 100 µl of internal standard 2-octanol (4.2 mg/l in ethanol) was added to each calibration solution, which was then submitted to SPE extraction and GC-MS analysis as described for the samples. Calibration curves were obtained using Chemstation software (Agilent Technologies, Inc.) by linear regression, plotting the response ratio (analyte peak area divided by internal standard peak area) against concentration ratio (added analyte concentration divided by internal standard concentration).

### 2.7 RNA extraction and reverse transcriptase quantitative PCR

Total RNA was extracted from ~200 mg of ground berry pericarp (pulp and skin) using the Spectrum Plant Total RNA kit (Sigma-Aldrich). Some modification to the protocol was performed according to Fasoli et al. (2012). RNA quality and quantity were determined using a Nanodrop 2000 spectrophotometer (Thermo Fisher Scientific) and DNA was removed with DNase I (Thermo Fisher Scientific). The cDNA synthesis and qRT-PCR analysis were carried out as previously described (Massonnet et al., 2017). Each expression value was measured in triplicate and normalized to the internal control *VvUBIQUITIN1*. The primer sets are listed in **Table S2**. Amplification efficiency and standard error values were calculated as previously described (Zenoni et al., 2020).

### 2.8 Statistical analysis

For the metabolites analysis, peak area values presented in the data matrix were Pareto scaled and centered and then submitted to an unsupervised principal component analysis (PCA) through SIMCA 13.0 (Umetrics). Supervised multivariate statistical analysis were carried out by clustering the samples based on indications inferred by PCA or previous information, and by performing an Orthogonal Bidirectional Partial Least Square-Discriminant Analysis (O2PLS-DA). The models were validated by performing a CV-ANOVA and a permutation test (400 permutations). The stilbenes and flavonols metabolite families were further investigated applying hierarchical clustering analyses (HCA) and Heatmap presentations, using Pearson correlation coefficient and Ward as clustering algorithm employing R (version 4.0.3) within the RStudio (version 1.3.1093) platform. For specific metabolites of interests, a one-way ANOVA between groups was performed by using SPSS software and Tukey’s test as post-Hoc (p<0.05).

As for the VOCs detected, the compound concentrations were auto-scaled (mean centered and divided by the standard deviation of each variable) and analyzed applying unsupervised PCA. A *t*-test was additionally applied on the VOCs families concentrations in the different conditions (p<0.05).

## 3 Results

### 3.1 Postharvest dehydration under different temperature conditions

**Figure 1:**
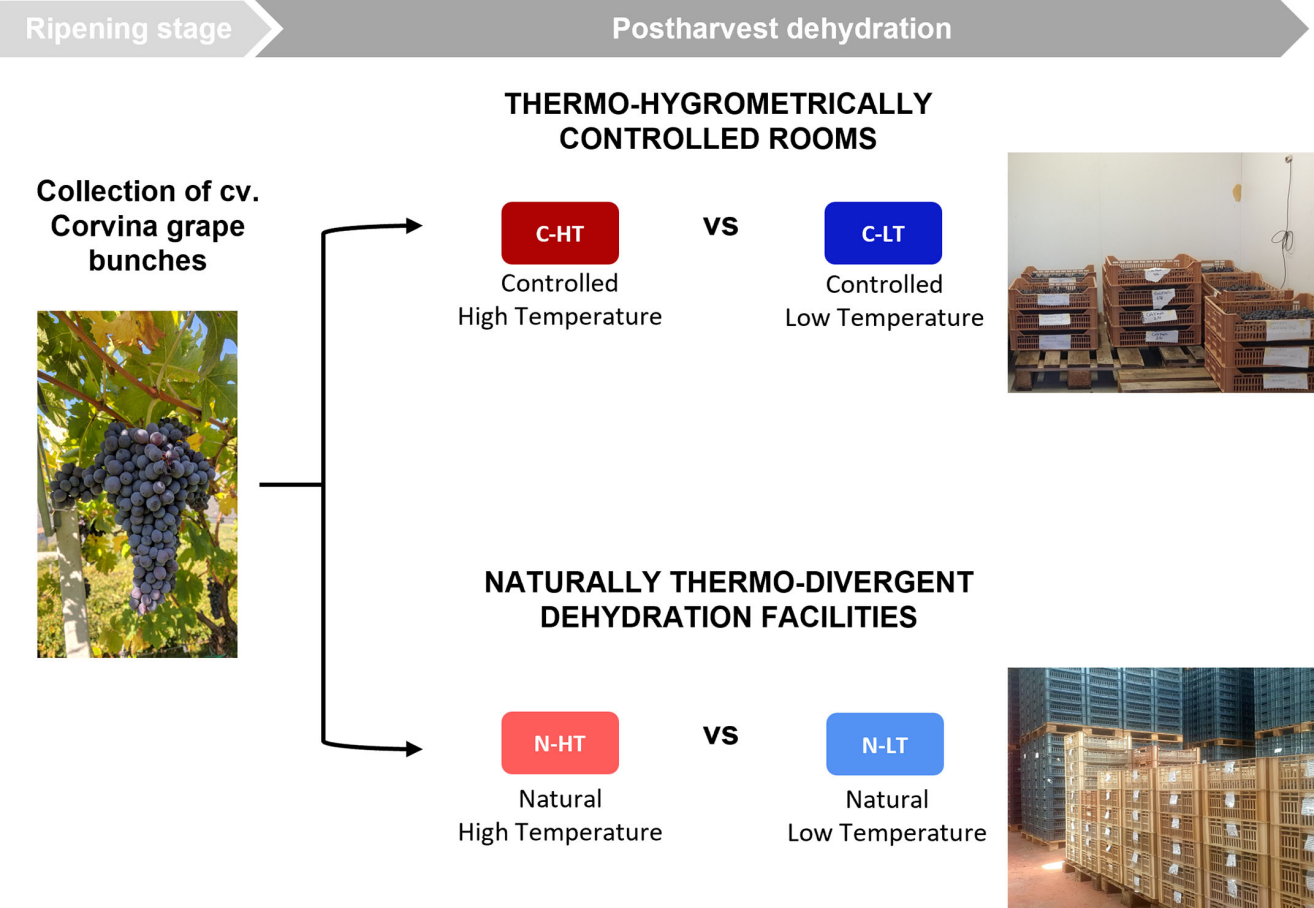
Overall experimental workflow. Grape bunches of cv. Corvina were collected at ripening stage. To evaluate the temperature effect on postharvest dehydration process, grape withering was conducted under different temperature conditions in two thermo-hygrometrically controlled rooms (C-HT; red vs C-LT; blue) and in two naturally thermo-divergent dehydration facilities (N-HT; light red vs N-LT; light blue).

**Figure 2:**
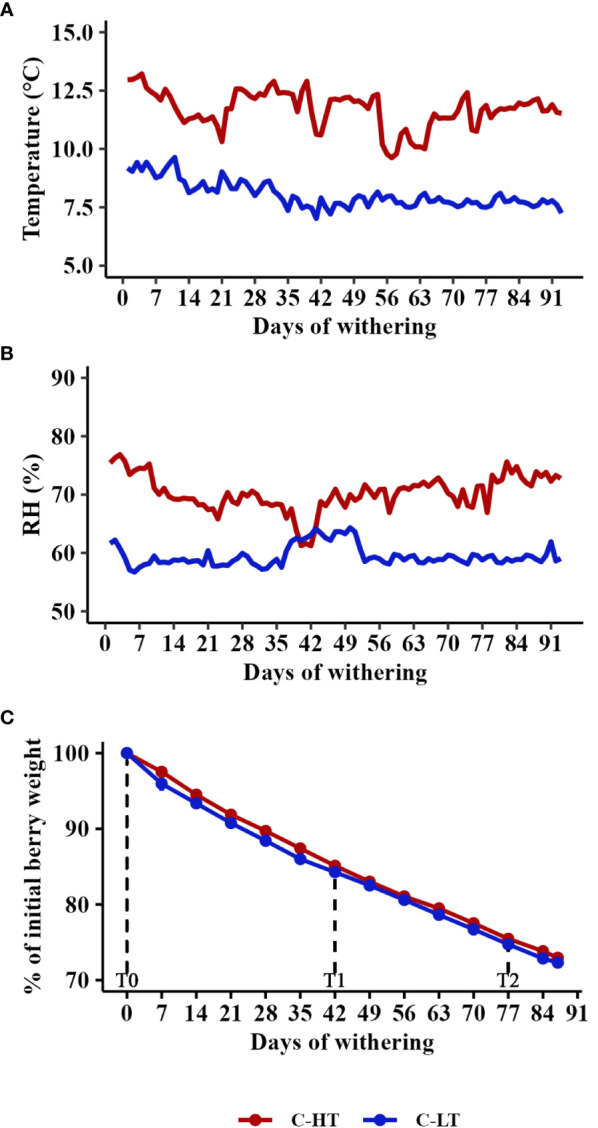
Postharvest dehydration in the thermo-hygrometrically controlled rooms. **(A)** Temperature and **(B)** Relative humidity (RH) in the C-HT and C-LT rooms during withering. **(C)** Weight loss kinetics of berries during withering in the C-HT and C-LT rooms reported as mean value ± SD of three biological replicates. For technological, metabolic and molecular analyses, grapes were collected during the withering processes at signed sampling time points corresponding to harvest time (T0), to ∼15% weight loss (T1) and to the end of withering (T2).

In order to dissect the effect of the temperature from that of the dehydration rate on grapes during withering we propose the experimental workflow described in **Figure 1**. Grape bunches of cv. Corvina were harvested and placed in two dedicated rooms at different monitored thermo-hygrometric conditions. The two rooms were set at different temperatures (average delta = 3.6 ± 0.5°C), while the relative humidity (RH) was adjusted to maintain the same grape water loss (WL) rate in both conditions. Thus, we defined a controlled high temperature (12 ± 0.5°C) and high RH (70 ± 3%) room (C-HT) and a controlled low temperature (8 ± 0.5 °C) and low RH (60 ± 2%) room (C-LT; **Figures 2A, B**; **Figure S1**). RH adjustments allowed to obtain the 28% of WL in 87 days in both C-HT and C-LT rooms (**Figure 2C**). Berry samples were collected at harvest (T0), at ∼15% weight loss (42 days after harvest, T1) and at ∼25% weight loss (77 days after harvest, T2) and further characterized.

Despite the pH values increased under both settings, TSS and TA were higher in grapes withered in the C-LT room (**Table 1**). The higher TA likely reflected a higher level of malic acid because an equal concentration of tartaric acid was revealed (**Table S3**).

**Table 1:** Total soluble solids (TSS), total acidity (TA) and pH measured in grapes dehydrated in the C-HT and C-LT rooms at sampling time points (T0, T1 and T2).

Parameter	T0			T1						T2					
				C-HT			C-LT			C-HT			C-LT		
**TSS (°Brix)**	22.07	±	0.15	25.30	±	0.11	26.63	±	0.25	28.53	±	0.06	31.20	±	0.25
**TA (g/L tartaric ac.)**	5.67	±	0.14	5.83	±	0.07	6.00	±	0.12	6.00	±	0.00	6.33	±	0.12
**pH**	3.21	±	0.02	3.37	±	0.03	3.34	±	0.01	3.53	±	0.04	3.48	±	0.01

*n*=3 ± SD. SD, Standard Deviation.

**Figure 3:**
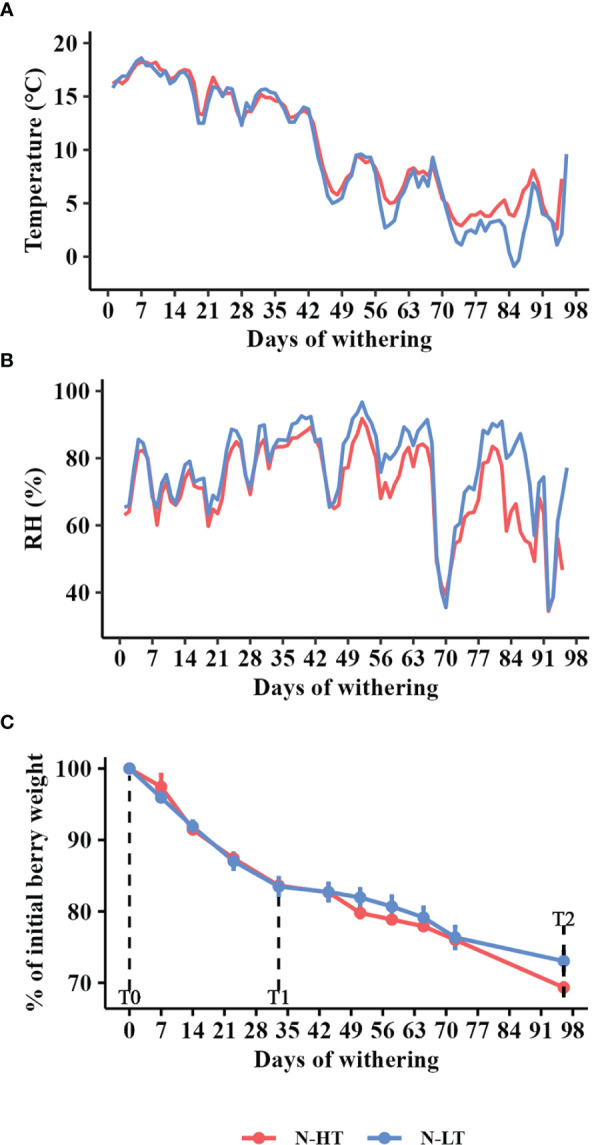
Postharvest dehydration in the naturally thermo-divergent N-HT and N-LT withering facilities. **(A)** Temperature and **(B)** Relative humidity (RH) registered in the facilities during withering. **(C)** Weight loss kinetics during withering in the N-HT and N-LT facilities reported as mean value ± SD of three biological replicates. For technological, metabolic and molecular analyses, grapes were collected during the withering processes at signed sampling time points corresponding to harvest time (T0), to ∼15% weight loss (T1) and to the end of withering (T2).

The temperature effect on cv. Corvina berries dehydration was also evaluated monitoring the process in two unconditioned dehydration rooms featuring the natural environmental conditions of two geographical locations, one located on a sunlight exposed hill side (N-HT) and the other located in a plain area typically characterized by the presence of fog (N-LT). Temperature and RH were monitored every two hours and daily average parameters were plotted (**Figures 3A, B**). Temperature trends of the two facilities were similar and varied within 10°C and 20°C during the first withering period (October-November). Only starting from the 45^th^ day of withering the temperature gradually decreased and started to diverge between the two processes (**Figure 3A**). As expected, the lowest temperature was detected at the N-LT facility where we also recorded a greater temperature span (6 to 7°C) in comparison to the N-HT facility (2 to 3°C; **Figure S2**). Likewise, RH was quite comparable between the two conditions during the first two months and diverged throughout the rest of the process registering the lowest value at the N-HT facility (**Figure 3B**).

Differently from the controlled rooms, divergent withering kinetics were registered at the two facilities (**Figure 3C**). In fact, given that both withering processes were carried out for 96 days, final WL values ended up being 27% and 31% in N-LT and N-HT conditions, respectively. Similarly to the controlled conditions, pH values did not change during the N-LT and N-HT dehydration processes, while TSS and TA progressively increased. However, subtly higher TSS and TA values were registered at higher temperature at natural compared to controlled withering conditions (**Table 2**).

**Table 2:** Total soluble solids (TSS), total acidity (TA) and pH measured in grapes dehydrated in the N-HT and N-LT facilities at sampling time points T0, T1 and T2).

Parameter	T0			T1						T2					
				N-HT			N-LT			N-HT			N-LT		
**TSS (°Brix)**	20.83	±	0.15	24.13	±	0.21	23.93	±	0.12	28.70	±	0.20	27.77	±	0.29
**TA (g/L tartaric ac.)**	7.25	±	0.00	7.08	±	0.08	7.62	±	0.13	8.20	±	0.10	8.00	±	0.00
**pH**	2.73	±	0.02	2.83	±	0.01	2.80	±	0.01	2.89	±	0.02	2.84	±	0.01

*n*=3 ± SD. SD, Standard Deviation.

### 3.2 Non-volatile metabolite profile changes during postharvest dehydration under different temperatures

**Figure 4:**
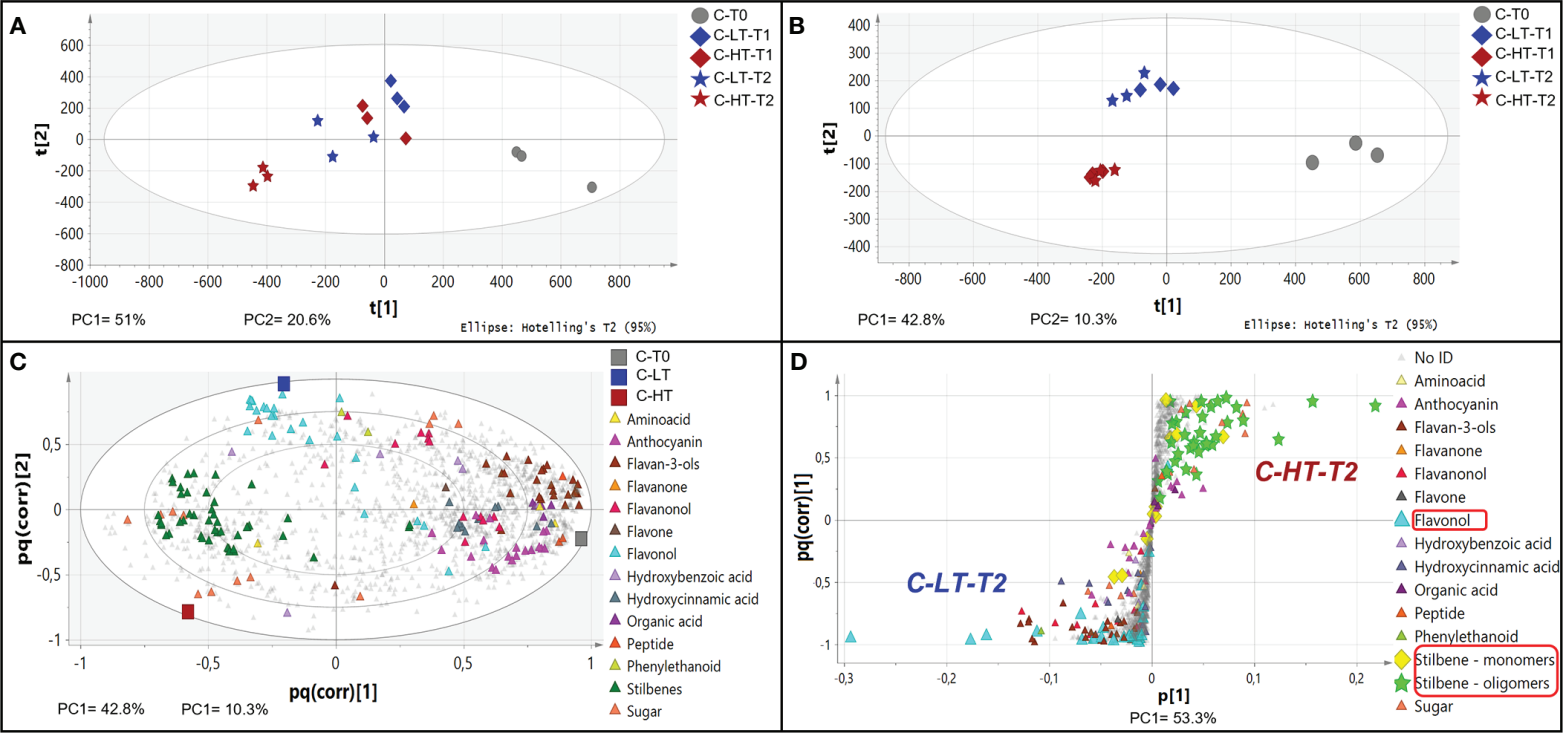
Non-volatile metabolite profile of grapes dehydrated in the C-HT and C-LT rooms. **(A)** PCA score scatter plot of non-volatile metabolites from berries collected during postharvest dehydration under C-HT and C-LT conditions. **(B)** O2PLS-DA score scatter plot highlighting a good sample clustering based on the withering conditions. **(C)** O2PLS-DA loading plot showing metabolites (triangles) featuring the mature phase (T0) and metabolites featuring drying berries collected in the C-HT and C-LT rooms. For this analysis, the T1 and T2 samples were grouped together. **(D)** OPLS-DA S-loading plot showing metabolites (triangles) correlating with the high or the low-temperature-condition at the end of the process (T2). Concerning stilbenes, monomeric forms are highlighted with yellow diamonds, whereas oligomers are shown as green stars.

**Figure 5:**
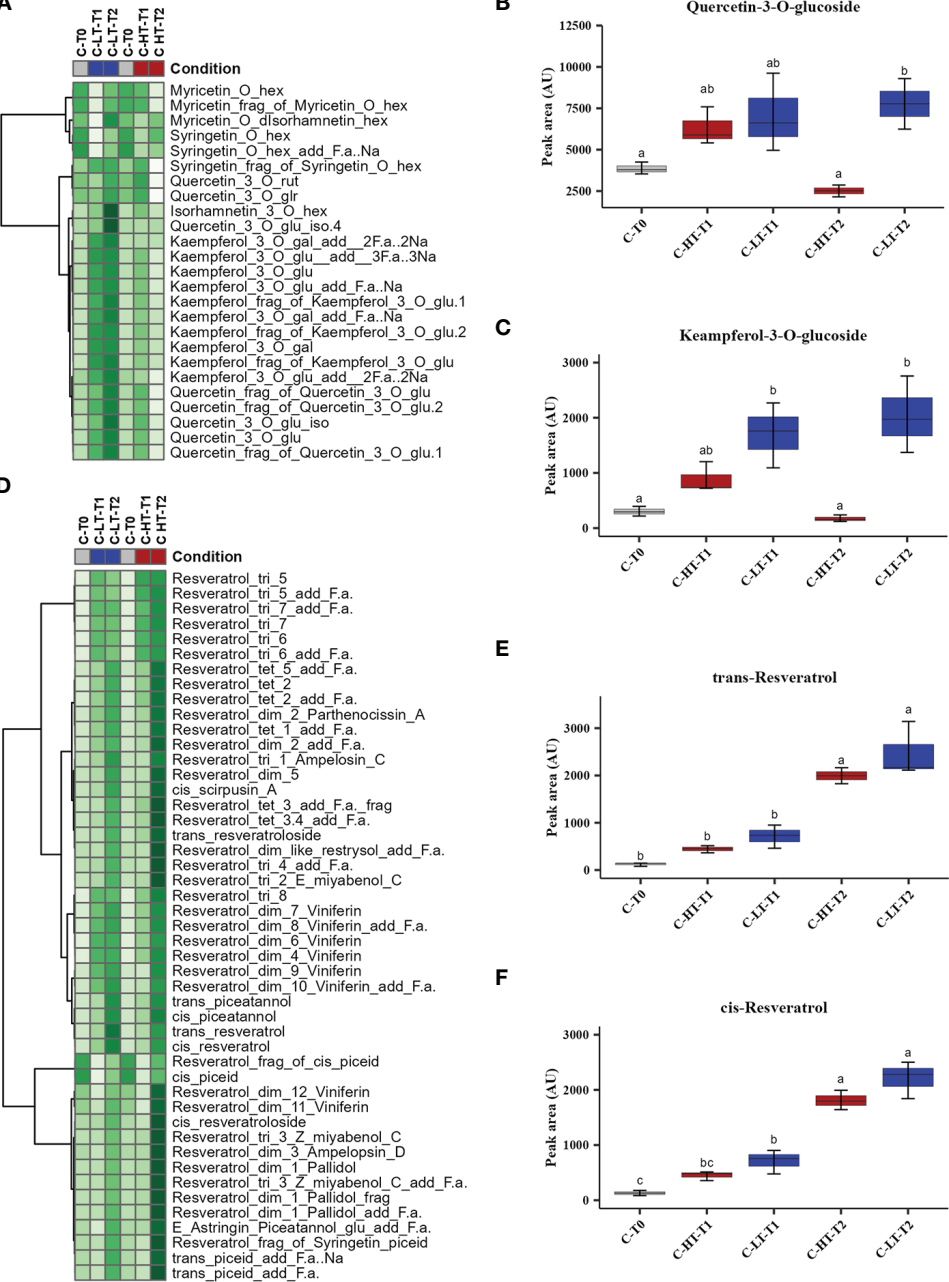
Variation of stilbenes and flavonols in grapes dehydrated in the C-HT and C-LT rooms. **(A)** Heatmap and Hierarchical Cluster Analyses performed on flavonols scaled peak areas (AU) measured at harvest (T0) and during withering (T1 and T2) under C-HT and C-LT conditions. Green and white colors represent high and low peak area values, respectively. Pearson correlation coefficient was employed to group the different metabolites, using average as clustering algorithm. Abbreviations: add=adduct; frag=fragment; f.a.=formic acid; gal=galactoside; glr=glucuronide; glu=glucose; hex=hexoside; iso=isotope; rut=rutinoside. **(B)** Quercetin-3-O-glucoside and **(C)** kaempferol-3-O-glucoside accumulation during withering in high and low temperature conditions. Lowercase letters represent statistically significant difference based on ANOVA and Tukey post-hoc test with p ≤ 0.05. **(D)** Heatmap and Hierarchical Cluster Analyses performed on stilbenes. **(E)** Trans-resveratrol and **(F)** cis-resveratrol accumulation during withering in high and low temperature conditions. Lowercase letters represent statistically significant difference based on ANOVA and Tukey post-hoc test with p ≤ 0.05. Abbreviations: add=adduct; dim=dimer; frag=fragment; f.a.=formic acid; gal=galactoside; glr=glucuronide; glu=glucose; hex=hexoside; iso=isotope; rut=rutinoside. tri=trimer; tet=tetramer. Gray colour indicates grapes at harvest (T0); blue colour indicates the C-LT condition; red colour indicates the C-HT condition.

The impact of temperature in the C-HT and C-LT dehydration rooms on the metabolic profile of the berries collected at T0, T1 and T2 was investigated by an untargeted UPLC-HRMS approach. The data processing through Progenesis QI generated a data matrix of 1585 rt/mz features, 182 of which were putatively identified (**Supplementary File 1**). The detected metabolites belonged to the class of amino acids, anthocyanins, flavan-3-ols, flavanonols, flavones, flavonols, hydroxybenzoic and hydroxycinnamic acids, organic acids, peptides, phenylethanoids, stilbenes and sugars. The data matrix was initially explored through an unsupervised PCA analysis (**Figure 4A**) that revealed a substantial distribution of the samples by withering time and C-HT/C-LT conditions along the PC1, explaining the 51% of the total variance. The PC2 explained the 20.6% of the total variance and appeared to discriminate samples mainly by the different withering condition. The PC1-PC2 plot showed that the samples of the two conditions were weakly separated at T1, whereas could be told apart at T2. In order to better highlight the effects of the withering condition, a O2PLS-DA supervised analysis was performed by superimposing the withering conditions as classes. The final model showed good sample clustering in the scatter plot (**Figure 4B**) and allowed to visualize the metabolites characterizing the specific classes in the loading plot (**Figure 4C**). Anthocyanins and flavan-3-ols better correlated with the harvest stage (T0) that, on the contrary, was poorly characterized by stilbenes. Interestingly, flavonols were associated with samples withered at low temperature (C-LT). Based on this information, an OPLS-DA was performed only on samples collected at T2 to emphasize any metabolic difference by withering condition at the end of the process (**Figure 4D**). The loading plot showed a strong correlation of stilbene oligomers, including dimers, trimers and tetramers with the C-HT condition, while several flavonols were highly associated with the C-LT condition. Due to their great correlation with the withering conditions, further investigations were carried out on the flavonol and stilbene classes of compounds (**Figure 5**). Hierarchical clustering analysis showed that flavonols were accumulated at T1 in both withering conditions and continued to increase at T2 in the berries exposed to low temperature, whereas a reduction was observed at T2 when exposed to high temperature (**Figure 5A**). This trend was well exemplified by the flavonols quercetin-3-O-glucoside and kaempferol-3-O-glucoside (**Figures 5B, C**). Concerning stilbenes, we observed the accumulation of these compounds to be a typical feature of the end of withering (T2) in both conditions. However, the polymerization degree diverged depending on the temperature conditions (**Figure 5D**) and higher stilbene complexity was found associated to HT-T2 grapes. Interestingly the stilbene monomers trans- and cis-resveratrol, representing the direct products of the key biosynthetic enzyme stilbene synthases, were found in greater amount in the LT-T2 grapes (**Figures 5E, F**).

### 3.3 Effect of withering temperature on the volatile profile in withered grapes and derived wines

**Figure 6:**
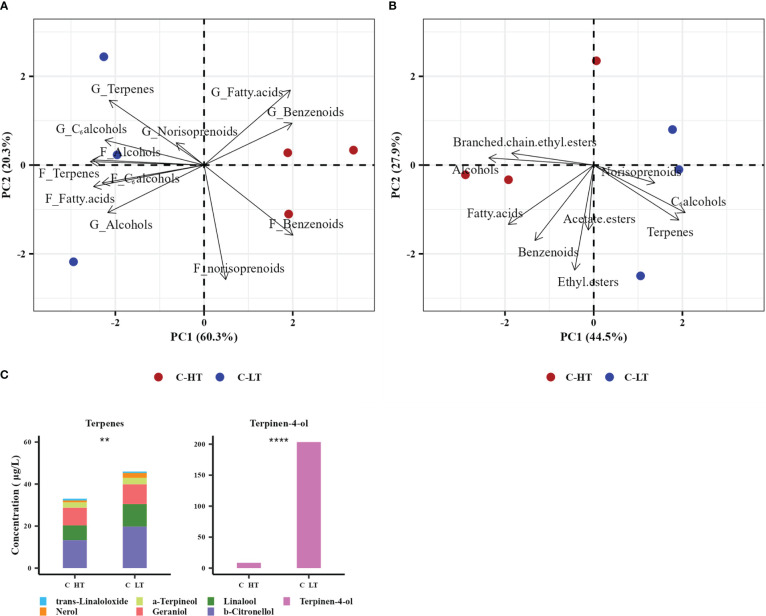
Aromatic profile of grapes dehydrated in the C-HT and C-LT rooms and in the derived wines. **(A)** PCA biplot of grape samples withered in the different rooms (circles) and the free VOCs (prefix F) and glycosidic precursors (prefix G) families identified in them (arrows). **(B)** PCA biplot of the derived wine samples (circles) and the free VOCs families identified in them (arrows). **(C)** Bar plot of the 7 free terpenes concentration in wines derived from grape berries dehydrated in the C-HT and C-LT rooms. Specific terpene accumulation is represented in different colors. Terpinen-4-ol was graphically separated due to its eminently higher concentration in both wines. Asterisks represent statistically significant difference based on t-test (p ≤ 0.01 = **; p ≤ 0.0001 = ****). Red color indicates the C-HT condition; blue color indicates the C-LT condition.

At the end of the process, the grapes dried under both controlled conditions and the derived wines were analyzed in triplicate by SPE-GC-MS to determine their volatile profiles. In the grapes, 27 glycosidic precursors (8 benzenoids, 6 terpenes, 4 alcohols, 4 C6 alcohols, 3 fatty acids and 2 norisoprenoids) and 24 free volatile compounds (8 benzenoids. 5 terpenes, 4 C6 alcohols, 3 alcohols, 3 fatty acids and 1 norisoprenoid) were detected and quantified (**Table S4**). The samples were then analyzed applying an unsupervised PCA using the VOCs families as predictors (**Figure 6A**). The PC1 and 2 explained respectively the 60.3% and the 20.3% of the total variance and evidenced a clear separation of the withering conditions by the PC1 and replicate variability mainly by the PC2. Most of the identified VOC families were highly associated with grapes withered under low temperature (C-LT): free terpenes, C_6_ alcohols, alcohol and their glycosidic precursors along with glycosidic norisoprenoids and free fatty acids. Interestingly, both free and glycosidic benzenoids families were instead associated to samples withered under high temperature (C-HT).

The volatile profile of the wines analyzed by SPE-GC-MS revealed 42 identified free VOCs (8 terpenes, 8 benzenoids, 6 alcohols, 5 ethyl esters, 4 C6 alcohols, 3 acetate esters, 3 fatty acids, 3 norisoprenoids and 2 branched-chain ethyl esters; **Table S5**) and was further investigated by unsupervised PCA. PC1 (explaining 44.5% variance) showed a better separation of the withering conditions compared to PC2 (explaining 27.9% variance) (**Figure 6B**). The C-LT wine samples were highly correlated with the terpenes, norisoprenoids and C6 alcohols families and, to a minor extent, with ethyl and acetate esters. Conversely, benzenoids, fatty acids, alcohols and branched chain ethyl esters were more associated with the C-HT wines. A graphic presentation of the free VOCs concentration is available in **Figure S3A**. To study the temperature effect on the compounds primarily deriving from the grape metabolism during withering, a further unsupervised PCA was performed using the single varietal VOCs as predictors (**Figure S3B**). Thus, only those compounds believed to be primarily of varietal origin and less affected by yeast activity during fermentation (i.e., terpenes, norisoprenoids, C_6_ alcohols and benzenoids) were considered in this analysis. The PC1 and 2 of the PCA model explained 59.3% and 19.1% of the total variance, respectively, and the biplot projection showed a clear separation of the wine samples by PC1 according to the withering condition, with most of the varietal VOCs clustering with the C-LT wines. On the other hand, the C-HT wines showed strong correlation only with benzaldehyde. The relative concentration of terpene compounds is reported in **Figure 6C**. Although the proportions among terpenes were analogous, the C-LT wines exhibited an overall higher content compared to the C-HT ones, suggesting that the effect exerted by temperature was comparable on each of the identified members of the terpene family.

These results suggest that different temperature conditions altered the wine VOC profile and that lower temperatures during grape withering could boost the overall terpenoid content in wines.

### 3.4 High temperature negatively affects the expression of quality-related genes during postharvest dehydration

**Figure 7:**
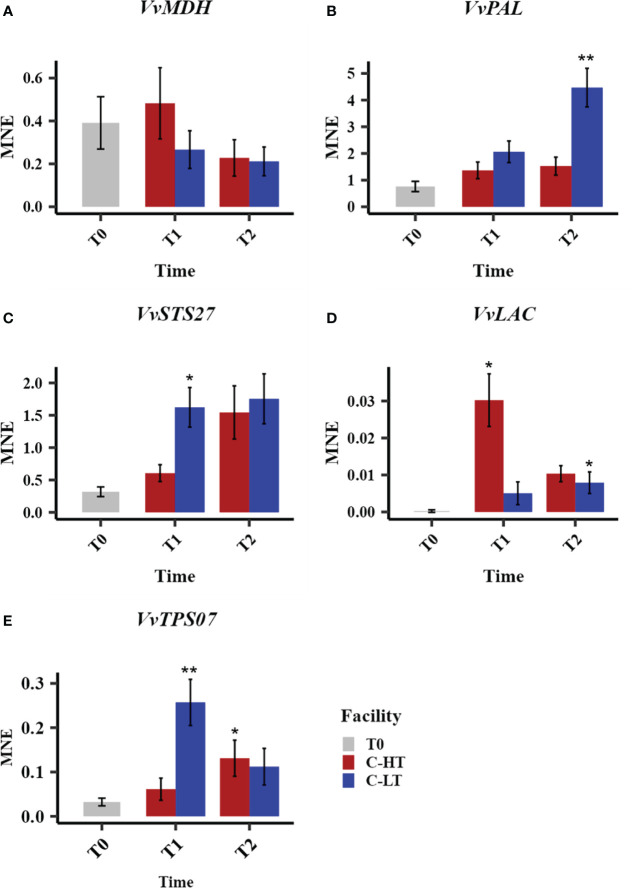
Relative expression of quality-related genes during withering in the C-HT and C-LT rooms. Expression profiles of *malate dehydrogenase* (*VvMDH*, **A**), *phenylalanine ammonia-lyases* (VvPAL, **B**), stilbene synthase (*VvSTS27*, **C**) *laccase* (*VvLAC*, **D**) and *terpene synthase* (*VvTPS07*, **E**) genes in grape sampled during withering in the C-HT and C-LT rooms. Error bars represent standard errors (n = 3). Blue colour indicates the C-LT condition; red colour the C-HT condition. Asterisks represent statistically significant difference between partially withered grapes (T1 and/or T2) and grapes at harvest (T0), based on t-test (p ≤ 0.05 = *; p ≤ 0.01 = **).

To study the effect of the different postharvest conditions (C-HT versus C-LT) on dehydrating berries at transcriptional level, we profiled the expression of some candidate genes, previously identified as players in the molecular mechanisms controlling the development of quality traits (Zenoni et al., 2020), at three time points (T0, T1 and T2) over withering (**Figure 7**).

The analysis of the expression level of a *malate dehydrogenase* (*VvMDH*), involved in malic acid degradation, revealed a downward trend throughout the dehydration process in both conditions, with a lower expression at T1 in berries dehydrated in C-LT room (**Figure 7A**). This fits with the progressive increase of the total acidity observed in both processes but greater when grapes are withered at lower temperature (**Table 1**, **Table S3**). We also profiled the expression of a *phenylalanine ammonia-lyase* (*VvPAL*), enzyme in charge of channeling phenylalanine into the phenylpropanoid pathway. The *VvPAL* expression level progressively increased during withering with a higher expression level (greater than 3-fold) in grapes withered in the C-LT room in comparison to the grapes processed in the C-HT room (**Figure 7B**). As expected, an upward expression pattern during withering was also described for the *stilbene synthase 27* (*VvSTS27*) that was previously reported to be a molecular marker of a slow grape dehydration process (Zenoni et al., 2020) (**Figure 7C**). Moreover, the lower temperature of the C-LT room determined a stronger and earlier induction of the expression of this gene compared to the other condition. The opposite trend was found for a laccase gene (*VvLAC*), that was also reported to be a marker generally upregulated during withering (Zenoni et al., 2020). In fact, *VvLAC* showed a sharp induction at T1 in the C-HT room, followed by a drop in expression thereafter. The withering conditions of the C-LT room instead determined only a slight induction throughout the process (**Figure 7D**). Finally, we examined the expression of a terpene synthase gene (*VvTPS07*) whose response to a lower temperature resembled that of *VvSTS27* at T1 that is significantly higher expression in the C-LT than in the C-HT room conditions (**Figure 7E**).

### 3.5 The temperature effect under natural conditions

In the N-HT and N-LT withering facilities, characterized by naturally divergent temperature conditions, dissimilar grape withering kinetics were determined (**Figure 3C**). In spite of that misalignment, the dehydrated berries at both conditions were compared for metabolomic changes at the end of the process and for the expression of *VvMDH*, *VvPAL*, *VvSTS27, VvLAC* and *VvTPS07*, at T0, T1 and T2 stages (**Table S1**).

**Figure 8:**
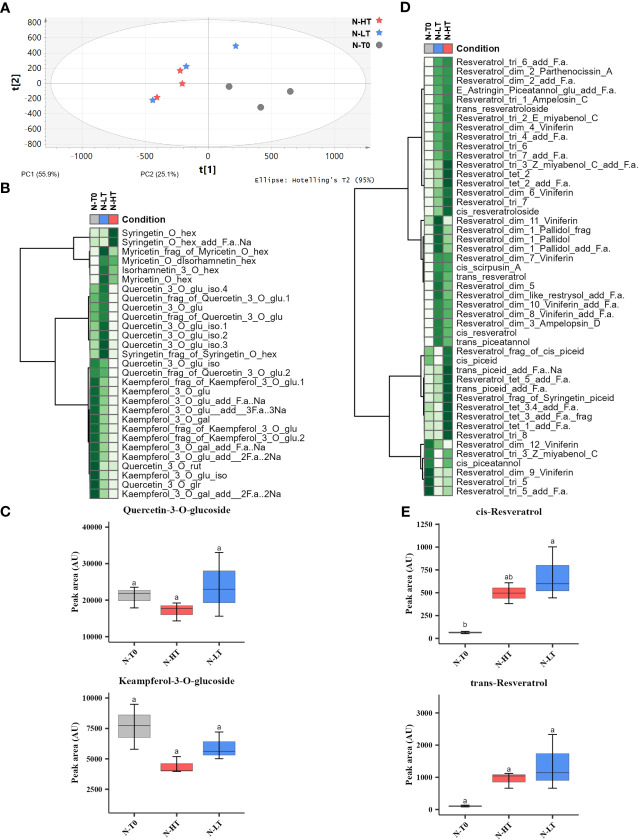
Metabolic profile and variation of stilbenes and flavonols in berries following dehydration in the N-HT and N-LT withering facilities. **(A)** PCA score scatter plot of non-volatile metabolites from berries collected at harvest (T0) and at the end of withering under N-HT and N-LT conditions. **(B)** Heatmap and Hierarchical Cluster Analyses performed on flavonols scaled peak areas (AU) measured at T0 and at the end of withering in the N-HT and N-LT conditions. Green and white colors represent high and low peak area values, respectively. Pearson correlation coefficient was employed to group the different metabolites, using average as clustering algorithm. Abbreviations: add=adduct; frag=fragment; f.a.=formic acid; gal=galactoside; glr=glucuronide; glu=glucose; hex=hexoside; iso=isotope; rut=rutinoside. **(C)** Quercetin-3-O-glucoside and kaempferol-3-O-glucoside accumulation during withering in high and low temperature conditions. Lowercase letters represent statistically significant difference based on ANOVA and Tukey post-hoc test with p ≤ 0.05. **(D)** Heatmap and Hierarchical Cluster Analyses preformed on the stilbenes. Abbreviations: add=adduct; dim=dimer; frag=fragment; f.a.=formic acid; gal=galactoside; glr=glucuronide; glu=glucose; hex=hexoside; iso=isotope; rut=rutinoside. tri=trimer; tet=tetramer. **(E)** Cis-resveratrol and trans-resveratrol accumulation during withering in high and low temperature conditions. Lowercase letters represent statistically significant difference based on ANOVA and Tukey post-hoc test with p ≤ 0.05. Gray colour indicates grapes at harvest (T0); light blue colour indicates the N-LT condition; light red colour indicates the N-HT condition.

By performing an untargeted UPLC-HRMS analysis we obtained a data matrix including 1584 rt/mz features, 181 of which were putatively identified (**Supplementary File 1**). The PCA analysis showed that the total explained variance was 81%, with the PC1 explaining the 55.9% and the PC2 the 25.1%, however, unlikely the PCA performed on C-HT and C-LT samples, it was not possible to clearly separate the berries withered at N-HT conditions from those at N-LT (**Figure 8A**). Nevertheless, focusing on flavonols and stilbenes families we found similarity to the trends observed for grapes withered under controlled conditions (**Figures 8B–E**). In details, the N-LT grapes presented an overall higher amount of flavonols, in comparison to the N-HT grapes. Besides, two distinct trends could be hypothesized: either higher accumulation in N-LT, as for quercetin and myricetin derivatives, or higher degradation in N-HT, as for kaempferol derivatives (**Figures 8B, C**). Moreover, the oligomeric form of the stilbenes was generally more prevalent in the N-HT grapes, while the stilbenes monomers cis- and trans-resveratrol were more abundant in the grapes withered at the lower temperatures (N-LT) (**Figures 8D, E**).

**Figure 9:**
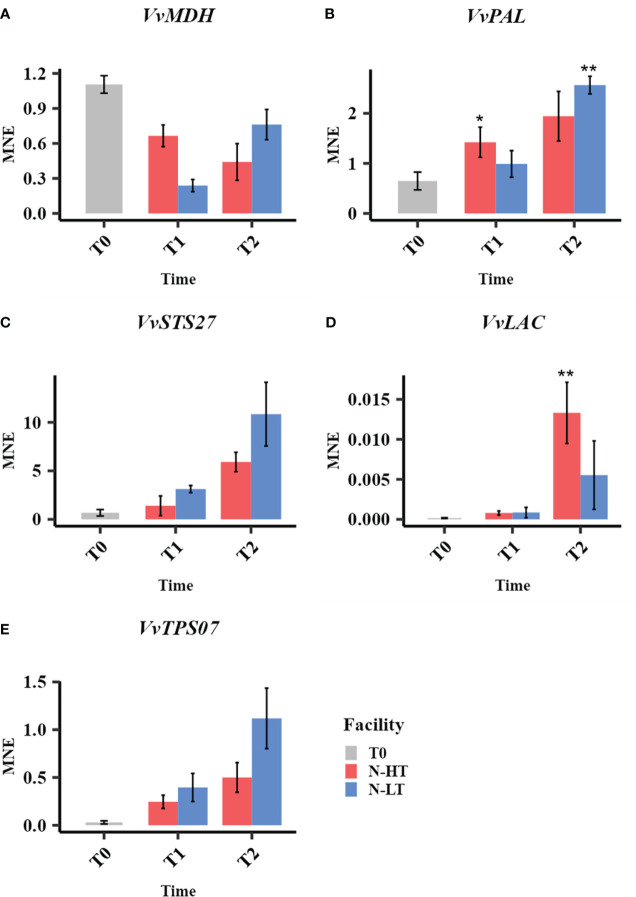
Expression profiles of malate dehydrogenase (*VvMDH*, **A**), phenylalanine ammonia-lyases (*VvPAL*, **B**), stilbene synthase (*VvSTS27*, **C**) laccase (*VvLAC*, **D**) and terpen synthase (*VvTPS07*, **E**) genes in grape sampled during withering in the N-HT and N-LT rooms. Error bars represent standard errors (n = 3). Light blue colour indicates the N-LT condition; light red colour the N-HT condition. Asterisks represent statistically significant difference between partially withered grapes (T1 and/or T2) and grapes at harvest (T0), based on t-test (p ≤ 0.05 = *; p ≤ 0.01 = **).

The analysis of candidate genes revealed similar expression trends to the one observed in the samples withered under controlled conditions. We found that the *VvMDH* expression level at the end of the process was lower than that registered at harvest (**Figure 9A**). We also observed that *VvMDH* was less expressed at T1 and more expressed at T2 in grapes withered at N-LT compared to N-HT. The expression of *VvPAL*, *VvSTS27*, *VvLAC* and *VvTPS07* showed an upward trend during withering under both natural conditions (**Figures 9B–E**). However, a differential expression level between the two different natural conditions was visible mainly at T2, likewise observed under controlled settings (**Figure 7**). This can be explained because major temperature differences between facilities were indeed observed only after the T1 sampling point. Notably, under natural conditions the higher induction of *VvSTS27* and *VvTPS07* at low temperature was maintained also at T2, while under controlled conditions, the temperature effect on the expression of the *VvSTS27* and *VvTPS07*, appeared strong at T1 and less effective at T2.

## 4 Discussion

### 4.1 A specific experimental design to dissect the temperature effect

The metabolic changes and the molecular events that characterize the grape postharvest dehydration process strictly depend on the genotype but also on the environmental parameters, in particular temperature and humidity, that typify the facilities where the grapes are placed to undergo withering (Costantini et al., 2006; Mencarelli et al., 2010; Zenoni et al., 2016). Despite the availability of many studies comparing processes conducted at different environmental conditions, including temperature variation, the effect of temperature on grapes placed in dehydrating room after harvest has not been conclusive. Besides the remarkable challenge of setting apart the temperature effect from other factors in experiments featuring a complexity of conditions (e.g., different grape varieties, environmental parameters, times, dehydration kinetics, etc.), the complication consists in the influence of the temperature on the grape water loss rate that in turn affects the berry transcriptional and metabolic reprograming occurring during the process (Zenoni et al., 2020). In this work, we analyzed the relationship between environmental conditions and chemical/molecular changes occurring in withering berries and took a step forward in exploring the effect of temperature excluding the puzzling effect of the dehydration rate. To do this we set up an experimental plan in which cv. Corvina grapes were dehydrated at two distinct temperatures while maintaining the same withering kinetics by daily adjustments of the relative humidity. Even though we could not completely rule out that such differences in relative humidity could have directly affected the berry metabolism, we assume that this is an unlikely scenario. Zhang and Keller (2017) postulated that increasing the relative humidity around bunches on the plant could impair the berry ripening metabolism as an effect of a reduced berry cuticular transpiration. Salvetti et al. (2016) showed that removing part of the humid air stacking around withering Corvina clusters resulted in a diversification of the grape microbiota. We did not analyze the microbiota of the grapes withered in the two conditioned room that may indeed be different and may have influenced the berry composition. However, except for specific cases (e.g., the *Botrytis cinerea* infection), the relationships between berry metabolism and the microbial consortium associated to postharvest grapes is far to be elucidated.

The conditioned facilities housing the grapes could not ensure a strict control of temperature that fluctuated within a range of 2°C depending on the external climatic condition (fall season). Nonetheless, the temperature difference between the two rooms was maintained in the range of 4 ± 1.0 °C for the whole period. The daily adjustments of the relative humidity in the two rooms were based both on daily measurements of the grape weight loss and the application of an empirical model. By this approach we obtained similar dehydration kinetics in the two rooms, allowing to attribute the observed grape compositional and molecular changes to the temperature difference. The grapes dehydrated at higher temperature featured slightly lower levels of sugars and titratable acidity (TA) which may be the result of the impact of the temperature on primary metabolism. The close relation between malic acid respiration and temperature is well known for ripening berries and few indications suggest that such relation may hold true also during the postharvest storage of the grapes (D’Onofrio et al., 2019). Tartaric acid was unaffected by temperature, strongly supporting that the differences in TA were entirely attributable to malic acid degradation. Interestingly, higher *MDH* gene expression level was detected in the grapes under C-HT conditions, indicating that an increased availability of malic acid degrading enzymes may account for its enhanced depletion.

Seeking to confirm the results obtained in the conditioned rooms, we set up a parallel trial placing the grapes in two unconditioned commercial facilities conjecturing they could hold different indoor temperatures in relation to their geographical locations. In the considered experimental year, the two commercial facilities showed very weak average daily temperature differences, that mainly occurred during the second half of the dehydration period. Despite this have certainly limited any differential response of berry metabolism, we could analytically highlight differences for few metabolite or molecular markers.

### 4.2 The influence of temperature on phenolic compounds

Several reports showed that changes in phenolic amounts and profile are part of the compositional rearrangement of the grapes when partially dehydrated after harvest and resulting from the simultaneous activity of biosynthetic and catabolic metabolic processes. Albeit highly dependent on the genotype and dehydration method, it was observed that most classes of compounds undergo a general decrease in absolute content possibly related to oxidation phenomena. Only in few cases, a clear induction of specialized biosynthetic branches leads to the accumulation of a specific class of compounds. Consistently, we observed a reduction of the level of total anthocyanin together with a strong increase in monomeric and oligomeric stilbenes in berries dehydrated under both temperature regimes. However, while anthocyanins were not apparently different between C-HT and C-LT, lower temperatures promoted the accumulation of the two primary products of STS (i.e., the monomeric trans-resveratrol and cis-resveratrol) and higher temperatures stimulated the increase of stilbene oligomeric forms. This result is consistent with the expression analysis of *PAL* and *STS*, which were additionally induced by the lower temperature conditions. Moreover, considering the putative involvement of LACs in producing dimers or higher degree phenolic oligomers – including stilbenes (Zenoni et al., 2016; Vezzulli et al., 2019; Zenoni et al., 2020; Pilati et al., 2021) – by oxidative polymerization, we speculate that the observed higher expression of a LAC gene at higher temperature conditions may denote the general higher stilbene complexity found in C-HT grapes containing greater amount of stilbene dimers, trimers and tetramers.

Among the phenolic compounds, the clear effect of the temperature on flavonols consisted in a general constant increase of kaempferol and quercetin derivatives throughout the whole C-LT process, and only a weak transient increase at C-HT conditions. Augmentation of flavonols, beyond the concentration effect, has been observed in grape berries undergoing postharvest dehydration. Bonghi et al. (2012) noted higher quercetin content in faster (by adjustments at higher temperature and lower RH) compared to slower dehydrating Raboso Piave berries, that however did not match the expression of a flavonol synthase (FLS) that showed higher expression in slow dehydration rate conditions. Net increments in the content of quercetin and kaempferol were also reported for Cesanese grape berries subjected to the different rate of dehydration at either 10 or 20°C (Bellincontro et al., 2009), with a higher increase observed at 20°C corresponding to the faster dehydration rate. Contrary to our findings, these studies showed that the increase in flavonol content was associated to higher temperatures. Noteworthy, higher temperatures were associated with greater dehydration rates that may have limited the time for the oxidative catabolism of these compounds. By dissecting the effect of temperature from the dehydration rate we were able to highlight that the low temperature condition may act in favor of the accumulation of flavonols. However, because we did not measure the expression of *FLS*, we cannot establish whether this was the result of an increased biosynthesis or of a reduced degradation.

The additional recorded studies showing significant changes (either increase or decrease) in the level of flavonols (Sanmartin et al., 2021) deal with different genotypes and quite different applied dehydration conditions likely implying the presence of dissimilar processes acting on the flavonol composition and that make any comparison with our study rather inconclusive.

### 4.3 Effect of temperature on volatile organic compounds

Mencarelli and Bellincontro (2020) reported that the generation of volatile compounds from amino acid and fatty acid catabolism is a common feature in grape berries dehydrated at relatively high temperatures. Conversely, lower temperatures seem more effective in preserving the varietal aromatic compounds including terpenoids and aldehydes. In all the reported studies, the different temperature conditions impacted the grape dehydration rate, and it was recently demonstrated that higher dehydration rates, obtained without temperature manipulation, dampen the typical postharvest transcriptomic program and determine a weaker induction of the terpene synthase genes (Zenoni et al., 2020). Thus, it is impossible to establish at which extent the observed effect on the grape VOCs was due to the difference in temperature regimes or in dehydration rates. In our study we compared different temperature conditions while maintaining similar dehydration rates and were thus able to show the direct effect of the temperature on the berry VOCs. C-LT condition increased terpenes and C_6_ alcohols content in grapes and wine, whereas C-HT favored esters and fatty acids content. Among the terpenes, the most significant change was recorded for terpinen-4-ol, a known marker of grape postharvest dehydration (Accordini, 2013; Negri et al., 2017) and, from our results, an indicator of grapes dehydrated at the lower temperature. Dehydration at low temperatures likely stimulated the terpene biosynthetic pathway directly as supported by the increased expression of in C-LT grapes.

Overall, our findings show that the temperature at which the grapes are dehydrated postharvest affects several berry metabolisms impacting the quality traits of the dehydrated grapes, hence of the resulting wines. We provided clear evidence of the positive effect of low temperatures in reducing the degradation of malic acid and favoring the accumulation of terpenes. Stilbenes metabolism was aroused by low temperature conditions with increased synthesis of stilbene monomers, whereas higher temperatures likely favored the formation of complex oligomeric forms. This result showcases the great impact that the temperature has not only on growth and ripening of the grapes but also on physiology and metabolism of the berry during postharvest dehydration.

Given that in real-life commercial conditions, higher temperature regimes are always associated to higher dehydration rates and shorter dehydration processes, the general impact of temperature on the final product should be re-evaluated considering both the direct effect on grape metabolism and the indirect effect that takes place through the modulation of the dehydration rate.

## Data availability statement

The original contributions presented in the study are included in the article/**Supplementary Material**. Further inquiries can be directed to the corresponding authors.

## Author contributions

GT and SZ designed the research. RS set up the two-rooms withering comparisons, collected berry samples and performed technological analyses, performed the micro-vinifications. AA followed the grape withering in natural conditions. AA, ED’I and RS performed the molecular analyses. MC performed metabolomic analysis of non-volatile metabolites. GL and MU performed the metabolomic analysis of aroma compounds. RS, GT, AA, SZ and MF interpreted data and wrote the manuscript. All authors contributed to the article and approved the submitted version.
