# Supplementary material for: Temperature affects organic acid, terpene and stilbene metabolisms in wine grapes during postharvest dehydration
Source: Front Plant Sci. 2023 Jan 30;14:1107954. doi: 10.3389/fpls.2023.1107954 (PMC9923099; doi:10.3389/fpls.2023.1107954)
Supplement: Supplementary file 1 [file DataSheet_1.docx]

Supplementary Material


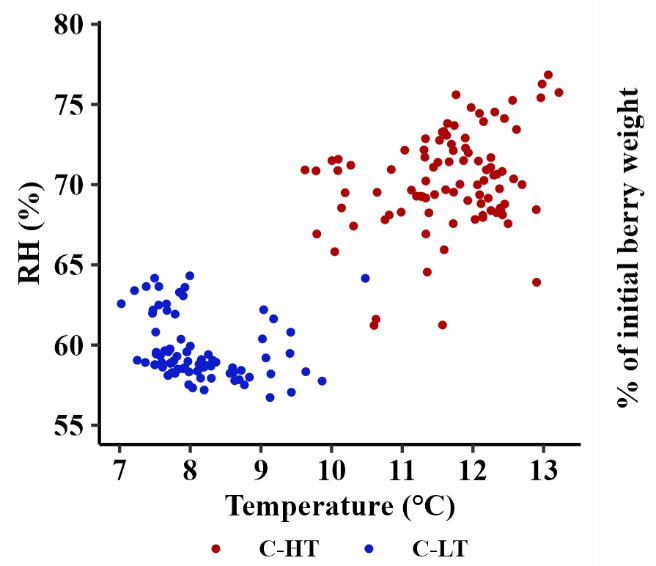


**Supplementary Figure 1.** **Postharvest dehydration conditions in the thermo-hygrometrically controlled rooms**. Temperature and relative humidity (RH) set in the C-HT and C-LT rooms during withering. Each dot represents the daily average of the set conditions.

**
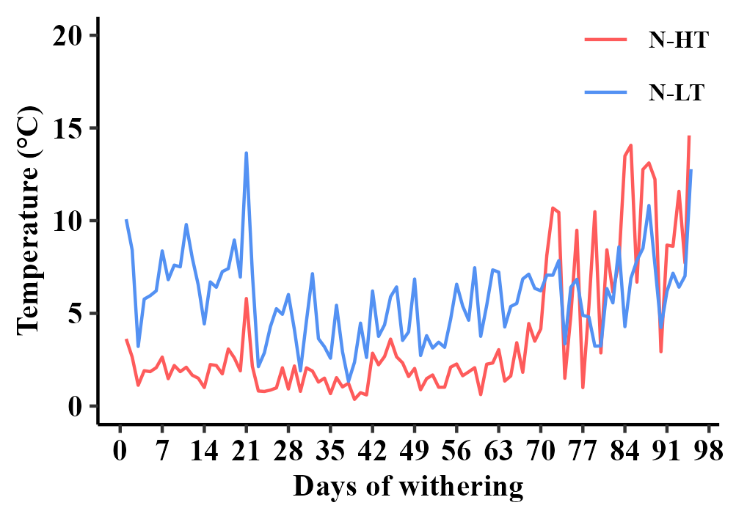
**

**Supplementary Figure 2: Thermal excursion in the naturally thermo-divergent N-HT and N-LT facilities during withering dehydration.** The thermal excursion was calculated as the difference between the maximal and minimal daily temperature.


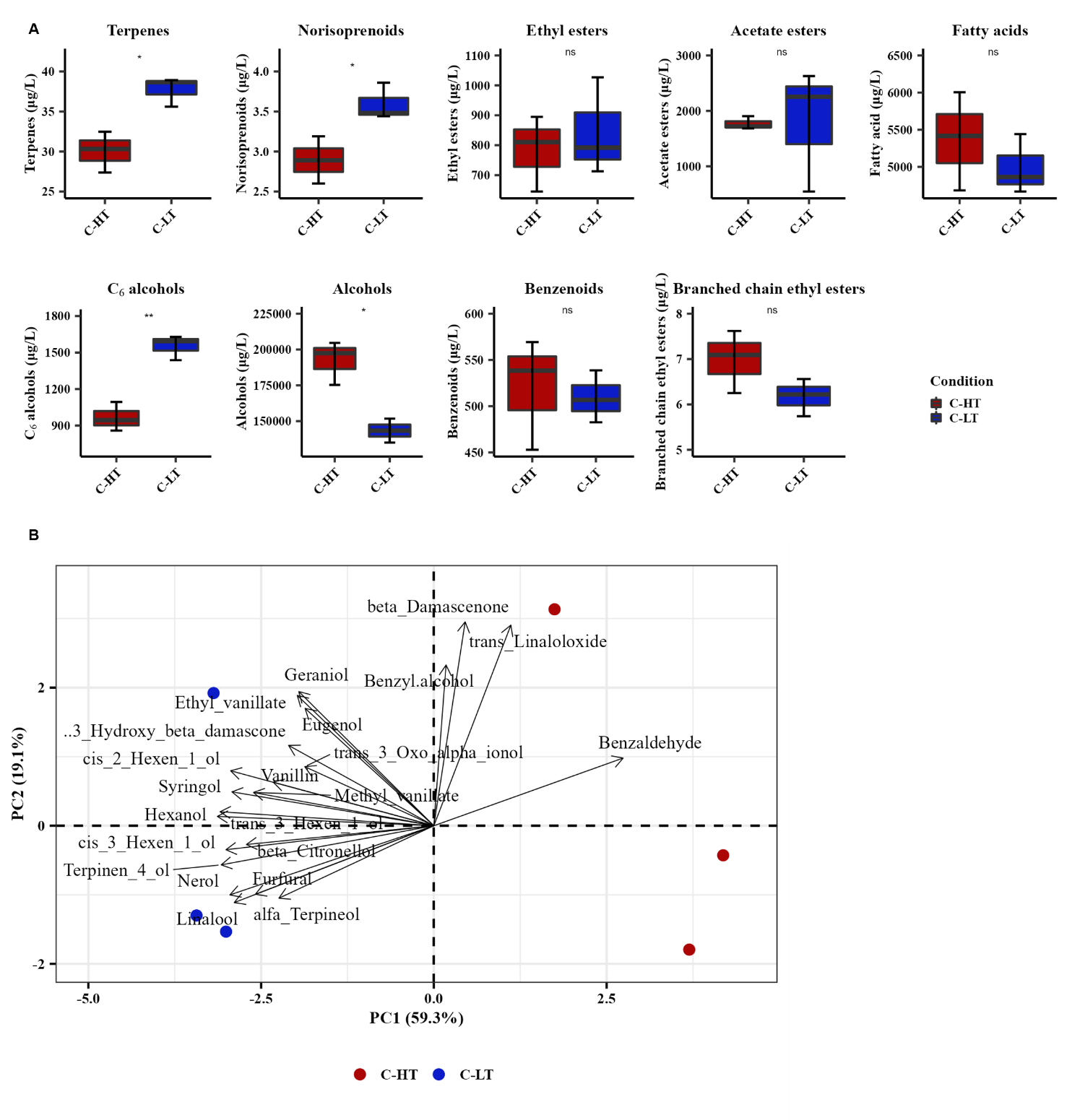


**Supplementary Figure 3: Temperature effect on wine aromatic profile during withering. (A)** Free VOCs concentration in wine derived from berries dehydrated at high and low temperature conditions. Asterisk represents significant differences (t-test; p< 0.05); ns, non-significant. (**B**) PCA biplot of the derived wine samples (circles) and the free varietal VOCs identified in them (arrows).

**Supplementary Table 1: Details of sample collection**.

| **Experimental details** | **Sampling details** | **T0** | **T1** | **T2** |
| --- | --- | --- | --- | --- |
| **Controlled conditions** | **Sampling data** | Sptember 9th | October 30th | December 7th |
|  | **Withering day** | 0 | 42 | 77 |
|  | **WL% C-HT** | 0 | 15 | 25 |
|  | **WL% C-LT** | 0 | 16 | 25 |
| **Natural conditions** | **Sampling data** | October 3th | November 5th | January 7th |
|  | **Withering day** | 0 | 33 | 96 |
|  | **WL% N-HT** | 0 | 16 | 31 |
|  | **WL% N-LT** | 0 | 17 | 27 |

**Supplementary Table 2**: **List of primer set used**

| **GENE** | **GENE ID** | **SEQUENCE 5'-3'** | **Reference** |
| --- | --- | --- | --- |
| ***VvMDH*** | VIT_07s0005g03360 | Fw - TGAAGAGGCATTTCTGCAGTT | this work |
|  |  | Rev - AAGATGCAAAAACATGGGAGAA |  |
| ***VvPAL*** | VIT_16s0039g01320 | Fw - GTGATGGAGAGCATGAGCAA | this work |
|  |  | Rev - GCACCACCTTGTTTGGTTCT |  |
| ***VvSTS27*** | VIT_16S0100G00990 | Fw - ATTCTGCAGGTGCCATTGCA | Zenoni et al. (2020) |
|  |  | Rev - ATACCAAGTGGGTCAAAAGCC |  |
| ***VvLAC*** | VIT_18s0001g01280 | Fw - TCACAGTGATTGGACCCGAA | Zenoni et al. (2020) |
|  |  | Rev - AATCAGAGGCATTGGGGTCA |  |
| ***VvTPS07*** | VIT_18s0001g04280 | Fw - TACCTTAGCCGCCCCTAATG | Zenoni et al. (2020) |
|  |  | Rev - AGCACTACCAAACCGAGAGA |  |
| ***VvUBIQUITIN1*** | VIT_16s0098g01190 | Fw - TCTGAGGCTTCGTGGTGGTA | Zenoni et al. (2020) |
|  |  | Rev - AGGCGTGCATAACATTTGCG |  |

**Supplementary Table 3:** Glucose+fructose, total acidity (TA) and tartaric acid measured by enzymatic kit in juice derived from cv. Corvina grape dehydrated in the C-HT and C-LT rooms at T2 (*n*=3 ± SD). SD, Standard Deviation.

| **Parameter** | **C-HT** | | | **C-LT** | | |
| --- | --- | --- | --- | --- | --- | --- |
| **Glucose+fructose (g/L)** | 291.56 | ± | 4.25 | 318.22 | ± | 7.02 |
| **TA (g/L tartaric ac.)** | 4.53 | ± | 0.06 | 4.93 | ± | 0.15 |
| **Tartaric acid (g/L)** | 2.17 | ± | 0.06 | 2.07 | ± | 0.09 |

**Supplementary Table 4:** Concentration (µg/L) of free VOCs and their glycosidic precursors in grapes dehydrated in the C-HT and C-LT rooms (*n*=3 ± standard deviation).

| **Compound** | **C-HT** | | | **C-LT** | | |
| --- | --- | --- | --- | --- | --- | --- |
| **Free VOCs** |  |  |  |  |  |  |
| **Alcohols** |  |  |  |  |  |  |
| Isoamyl alcohol | 181.05 | **±** | 42.09 | 383.94 | **±** | 55.41 |
| 1-Pentanol | 4.24 | **±** | 0.52 | 7.53 | **±** | 3.32 |
| Phenylethyl alcohol | 97.57 | **±** | 4.93 | 132.16 | **±** | 4.39 |
| **C6 alcohols** |  |  |  |  |  |  |
| 1-Hexanol | 224.74 | **±** | 15.48 | 257.17 | **±** | 15.64 |
| trans-3-Hexen-1-ol | 2.94 | **±** | 0.20 | 3.05 | **±** | 0.10 |
| cis-3-Hexen-1-ol | 12.47 | **±** | 0.79 | 20.01 | **±** | 1.17 |
| cis-2-Hexen-1-ol | 59.16 | **±** | 7.76 | 57.38 | **±** | 20.20 |
| **Terpenes** |  |  |  |  |  |  |
| Linalool | 1.06 | **±** | 0.17 | 1.81 | **±** | 0.24 |
| Nerol | 2.01 | **±** | 0.07 | 1.47 | **±** | 0.47 |
| Geraniol | 4.87 | **±** | 1.26 | 7.14 | **±** | 1.98 |
| Terpinen-4-ol | 34.41 | **±** | 1.17 | 130.37 | **±** | 23.92 |
| α-Terpineol | 0.80 | **±** | 0.18 | 1.08 | **±** | 0.19 |
| **Norisoprenoids** |  |  |  |  |  |  |
| 3-Oxo-α-ionol | 0.89 | **±** | 0.05 | 0.82 | **±** | 0.21 |
| **Benzenoids** |  |  |  |  |  |  |
| Eugenol | 1.94 | **±** | 0.24 | 1.30 | **±** | 0.96 |
| Furfural | 0.75 | **±** | 0.49 | 0.47 | **±** | 0.01 |
| Benzaldehyde | 13.80 | **±** | 0.54 | 7.67 | **±** | 7.37 |
| Benzyl alcohol | 349.31 | **±** | 30.71 | 207.37 | **±** | 62.22 |
| 2,6-Dimethoxy-phenol | 6.34 | **±** | 1.15 | 6.16 | **±** | 0.07 |
| Vanillin | 30.83 | **±** | 2.73 | 35.25 | **±** | 2.52 |
| Methyl-vanillate | 5.31 | **±** | 0.31 | 5.23 | **±** | 0.05 |
| Ethyl-vanillate | 135.69 | **±** | 13.57 | 159.91 | **±** | 3.44 |
| **Fatty acids** |  |  |  |  |  |  |
| 3-Methylbutanoic acid | 2.56 | **±** | 0.47 | 19.83 | **±** | 13.59 |
| Hexanoic acid | 280.41 | **±** | 4.64 | 367.96 | **±** | 16.65 |
| Octanoic acid | 136.32 | **±** | 28.47 | 203.61 | **±** | 24.82 |
| **Glycosidic precursors** |  |  |  |  |  |  |
| **Alcohols** |  |  |  |  |  |  |
| 1-Butanol | 1.76 | **±** | 0.93 | 3.35 | **±** | 0.73 |
| Isoamyl alcohol | 25.48 | **±** | 3.14 | 37.11 | **±** | 7.60 |
| 1-Pentanol | 1.29 | **±** | 0.37 | 1.24 | **±** | 0.16 |
| Phenylethyl qlcohol | 102.98 | **±** | 1.52 | 120.81 | **±** | 12.55 |
| **C6 alcohols** |  |  |  |  |  |  |
| 1-Hexanol | 44.40 | **±** | 4.01 | 54.50 | **±** | 3.72 |
| cis-3-Hexen-1-ol | 0.49 | **±** | 0.02 | 0.61 | **±** | 0.05 |
| trans-3-Hexen-1-ol | 6.33 | **±** | 0.52 | 8.16 | **±** | 0.50 |
| cis-2-hexen-1-ol | 3.49 | **±** | 0.10 | 4.36 | **±** | 0.12 |
| **Terpenes** | 0.00 |  |  |  |  |  |
| Linalool | 7.76 | **±** | 0.66 | 9.26 | **±** | 0.61 |
| Nerol | 7.39 | **±** | 0.73 | 5.39 | **±** | 4.03 |
| Geraniol | 16.68 | **±** | 1.86 | 25.57 | **±** | 1.24 |
| α-Terpineol | 1.33 | **±** | 0.19 | 1.34 | **±** | 0.27 |
| trans-Linaloloxide | 1.17 | **±** | 0.13 | 0.88 | **±** | 0.09 |
| cis-Linaloloxide | 0.95 | **±** | 0.03 | 1.12 | **±** | 0.11 |
| **Norisoprenoids** |  |  |  |  |  |  |
| 3-Hydroxy-β-damascone | 0.55 | **±** | 0.14 | 0.64 | **±** | 0.04 |
| 3-Oxo-α-ionol | 2.03 | **±** | 0.11 | 1.96 | **±** | 0.09 |
| **Benzenoids** |  |  |  |  |  |  |
| Furfural | 0.69 | **±** | 0.24 | 0.54 | **±** | 0.00 |
| Benzaldehyde | 2.15 | **±** | 0.31 | 1.30 | **±** | 1.21 |
| Benzyl Alcohol | 171.10 | **±** | 28.37 | 154.19 | **±** | 12.13 |
| Eugenol | 5.12 | **±** | 0.16 | 4.98 | **±** | 0.28 |
| 2,6-Dimethoxy-Phenol | 4.50 | **±** | 0.16 | 3.61 | **±** | 0.14 |
| Vanillin | 5.87 | **±** | 0.29 | 6.35 | **±** | 0.67 |
| Methyl-vanillate | 5.87 | **±** | 0.55 | 6.48 | **±** | 1.15 |
| Ethyl-vanillate | 19.82 | **±** | 1.74 | 8.99 | **±** | 2.74 |
| **Fatty acids** | 0.00 |  |  |  |  |  |
| 3-Methylbutanoic acid | 1.23 | **±** | 0.16 | 1.59 | **±** | 0.28 |
| Hexanoic acid | 18.51 | **±** | 3.28 | 10.90 | **±** | 2.26 |
| Octanoic acid | 134.64 | **±** | 15.68 | 119.46 | **±** | 24.71 |

**Supplementary Table 5:** Concentration (µg/L) of free VOCs identified in wines derived from grapes withered in the C-HT and C-LT rooms. (*n*=3 ± standard deviation).

| **Compound** | **C-HT** | | | **C-LT** | | |
| --- | --- | --- | --- | --- | --- | --- |
| **Free VOCs** |  |  |  |  |  |  |
| **Alcohols** |  |  |  |  |  |  |
| 1-Butanol | 170.33 | ± | 37.35 | 225.14 | ± | 43.13 |
| 2-Butanol | 80.04 | ± | 6.18 | 117.04 | ± | 21.07 |
| Isoamyl alcohol | 180344.8 | ± | 14242.67 | 129704.8 | ± | 12070.04 |
| 1-Pentanol | 128.93 | ± | 21.40 | 128.67 | ± | 5.59 |
| Methionol | 243.51 | ± | 39.17 | 200.25 | ± | 31.87 |
| Phenylethyl alcohol | 11488.61 | ± | 1022.47 | 7674.62 | ± | 412.57 |
| **Ethyl esters** |  |  |  |  |  |  |
| Ethyl butanoate | 183.69 | ± | 28.77 | 164.38 | ± | 40.09 |
| Ethyl hexanoate | 178.19 | ± | 47.89 | 303.97 | ± | 81.07 |
| Ethyl octanoate | 169.77 | ± | 47.51 | 148.77 | ± | 13.25 |
| Ethyl decanoate | 54.87 | ± | 3.32 | 52.68 | ± | 1.52 |
| Ethyl-3-hydroxybutanoate | 196.96 | ± | 25.99 | 174.09 | ± | 53.08 |
| **Branched chain ethyl esters** |  |  |  |  |  |  |
| Ethyl-2-methylbutanoate | 3.14 | ± | 0.20 | 2.43 | ± | 0.23 |
| Ethyl 3-methylbutanoate | 3.84 | ± | 0.50 | 3.74 | ± | 0.19 |
| **Acetate esters** |  |  |  |  |  |  |
| Isoamyl acetate | 479.64 | ± | 57.05 | 460.65 | ± | 117.47 |
| n-Hexyl acetate | 1258.90 | ± | 95.00 | 1319.45 | ± | 1013.91 |
| 2-Phenethyl acetate | 31.12 | ± | 3.09 | 29.84 | ± | 4.39 |
| **C6 alcohols** |  |  |  |  |  |  |
| 1-Hexanol | 893.10 | ± | 112.48 | 1422.95 | ± | 88.19 |
| trans-3-Hexen-1-ol | 8.50 | ± | 1.10 | 14.68 | ± | 1.30 |
| cis-3-Hexen-1-ol | 56.91 | ± | 4.55 | 105.45 | ± | 14.68 |
| cis-2-hexen-1-ol | 7.16 | ± | 1.67 | 10.33 | ± | 0.85 |
| **Terpenes** |  |  |  |  |  |  |
| Linalool | 7.04 | ± | 0.25 | 10.75 | ± | 1.14 |
| Nerol | 0.98 | ± | 0.06 | 2.33 | ± | 0.28 |
| Geraniol | 8.45 | ± | 1.38 | 9.43 | ± | 0.80 |
| Terpinen-4-ol | 8.50 | ± | 0.72 | 203.39 | ± | 21.45 |
| α-Terpineol | 2.47 | ± | 0.18 | 3.06 | ± | 0.52 |
| β-Citronellol | 13.29 | ± | 1.91 | 19.74 | ± | 1.75 |
| trans-Linaloloxide | 0.79 | ± | 0.15 | 0.64 | ± | 0.18 |
| **Norisoprenoids** |  |  |  |  |  |  |
| β-Damascenone | 0.70 | ± | 0.14 | 0.64 | ± | 0.05 |
| 3-Hydroxy-β-damascone | 0.05 | ± | 0.05 | 0.09 | ± | 0.02 |
| 3-Oxo-α-ionol | 1.60 | ± | 0.97 | 2.17 | ± | 0.15 |
| **Benzenoids** |  |  |  |  |  |  |
| Eugenol | 6.11 | ± | 1.00 | 7.09 | ± | 0.75 |
| Furfural | 0.48 | ± | 0.03 | 0.81 | ± | 0.19 |
| Benzaldehyde | 43.40 | ± | 9.17 | 10.46 | ± | 1.73 |
| Benzyl alcohol | 307.04 | ± | 35.17 | 301.00 | ± | 15.33 |
| 2,6-Dimethoxy-phenol | 6.11 | ± | 0.81 | 7.81 | ± | 0.58 |
| Vanillin | 4.30 | ± | 0.38 | 5.88 | ± | 1.23 |
| Methyl-vanillate | 6.26 | ± | 1.98 | 9.37 | ± | 1.58 |
| Ethyl-vanillate | 146.53 | ± | 23.08 | 166.94 | ± | 12.25 |
| **Fatty acids** |  |  |  |  |  |  |
| 3-Methylbutanoic acid | 431.86 | ± | 53.15 | 303.72 | ± | 13.55 |
| Hexanoic acid | 1634.48 | ± | 246.59 | 1644.37 | ± | 196.05 |
| Octanoic acid | 3301.41 | ± | 378.06 | 3042.52 | ± | 213.38 |

| **Compound** | **C-HT** | | | **C-LT** | | |
| --- | --- | --- | --- | --- | --- | --- |
| **Free VOCs** |  |  |  |  |  |  |
| **Alcohols** |  |  |  |  |  |  |
| 1-Butanol | 170.33 | ± | 37.35 | 225.14 | ± | 43.13 |
| 2-Butanol | 80.04 | ± | 6.18 | 117.04 | ± | 21.07 |
| Isoamyl alcohol | 180344.8 | ± | 14242.67 | 129704.8 | ± | 12070.04 |
| 1-Pentanol | 128.93 | ± | 21.40 | 128.67 | ± | 5.59 |
| Methionol | 243.51 | ± | 39.17 | 200.25 | ± | 31.87 |
| Phenylethyl alcohol | 11488.61 | ± | 1022.47 | 7674.62 | ± | 412.57 |
| **Ethyl esters** |  |  |  |  |  |  |
| Ethyl butanoate | 183.69 | ± | 28.77 | 164.38 | ± | 40.09 |
| Ethyl hexanoate | 178.19 | ± | 47.89 | 303.97 | ± | 81.07 |
| Ethyl octanoate | 169.77 | ± | 47.51 | 148.77 | ± | 13.25 |
| Ethyl decanoate | 54.87 | ± | 3.32 | 52.68 | ± | 1.52 |
| Ethyl-3-hydroxybutanoate | 196.96 | ± | 25.99 | 174.09 | ± | 53.08 |
| **Branched chain ethyl esters** |  |  |  |  |  |  |
| Ethyl-2-methylbutanoate | 3.14 | ± | 0.20 | 2.43 | ± | 0.23 |
| Ethyl 3-methylbutanoate | 3.84 | ± | 0.50 | 3.74 | ± | 0.19 |
| **Acetate esters** |  |  |  |  |  |  |
| Isoamyl acetate | 479.64 | ± | 57.05 | 460.65 | ± | 117.47 |
| n-Hexyl acetate | 1258.90 | ± | 95.00 | 1319.45 | ± | 1013.91 |
| 2-Phenethyl acetate | 31.12 | ± | 3.09 | 29.84 | ± | 4.39 |
| **C6 alcohols** |  |  |  |  |  |  |
| 1-Hexanol | 893.10 | ± | 112.48 | 1422.95 | ± | 88.19 |
| trans-3-Hexen-1-ol | 8.50 | ± | 1.10 | 14.68 | ± | 1.30 |
| cis-3-Hexen-1-ol | 56.91 | ± | 4.55 | 105.45 | ± | 14.68 |
| cis-2-hexen-1-ol | 7.16 | ± | 1.67 | 10.33 | ± | 0.85 |
| **Terpenes** |  |  |  |  |  |  |
| Linalool | 7.04 | ± | 0.25 | 10.75 | ± | 1.14 |
| Nerol | 0.98 | ± | 0.06 | 2.33 | ± | 0.28 |
| Geraniol | 8.45 | ± | 1.38 | 9.43 | ± | 0.80 |
| Terpinen-4-ol | 8.50 | ± | 0.72 | 203.39 | ± | 21.45 |
| α-Terpineol | 2.47 | ± | 0.18 | 3.06 | ± | 0.52 |
| β-Citronellol | 13.29 | ± | 1.91 | 19.74 | ± | 1.75 |
| trans-Linaloloxide | 0.79 | ± | 0.15 | 0.64 | ± | 0.18 |
| **Norisoprenoids** |  |  |  |  |  |  |
| β-Damascenone | 0.70 | ± | 0.14 | 0.64 | ± | 0.05 |
| 3-Hydroxy-β-damascone | 0.05 | ± | 0.05 | 0.09 | ± | 0.02 |
| 3-Oxo-α-ionol | 1.60 | ± | 0.97 | 2.17 | ± | 0.15 |
| **Benzenoids** |  |  |  |  |  |  |
| Eugenol | 6.11 | ± | 1.00 | 7.09 | ± | 0.75 |
| Furfural | 0.48 | ± | 0.03 | 0.81 | ± | 0.19 |
| Benzaldehyde | 43.40 | ± | 9.17 | 10.46 | ± | 1.73 |
| Benzyl alcohol | 307.04 | ± | 35.17 | 301.00 | ± | 15.33 |
| 2,6-Dimethoxy-phenol | 6.11 | ± | 0.81 | 7.81 | ± | 0.58 |
| Vanillin | 4.30 | ± | 0.38 | 5.88 | ± | 1.23 |
| Methyl-vanillate | 6.26 | ± | 1.98 | 9.37 | ± | 1.58 |
| Ethyl-vanillate | 146.53 | ± | 23.08 | 166.94 | ± | 12.25 |
| **Fatty acids** |  |  |  |  |  |  |
| 3-Methylbutanoic acid | 431.86 | ± | 53.15 | 303.72 | ± | 13.55 |
| Hexanoic acid | 1634.48 | ± | 246.59 | 1644.37 | ± | 196.05 |
| Octanoic acid | 3301.41 | ± | 378.06 | 3042.52 | ± | 213.38 |
